# Association of Implementing an Incentive Metric in the Oregon Medicaid Program With Effective Contraceptive Use

**DOI:** 10.1001/jamanetworkopen.2020.12540

**Published:** 2020-08-05

**Authors:** Maria I. Rodriguez, Thomas Meath, Jiaming Huang, Blair G. Darney, K. John McConnell

**Affiliations:** 1 Department of Obstetrics and Gynecology, Oregon Health & Science University, Portland; 2 Center for Health Systems Effectiveness, Oregon Health & Science University, Portland

## Abstract

**Importance:**

Reducing unintended pregnancy is a national public health priority. Incentive metrics are increasingly used by health systems to improve health outcomes and reduce costs, but limited data exist on the association of incentive metrics with contraceptive use.

**Objective:**

To evaluate whether an association exists between implementing an incentive metric and effective contraceptive use within the Oregon Medicaid program.

**Design, Setting, and Participants:**

In this state-level, claims-based cohort study, a comparative interrupted time series design was used to evaluate whether the implementation of an effective contraceptive use incentive metric on January 1, 2015, was associated with changes in contraceptive use among Oregon Medicaid adult enrollees when compared with commercially insured women. The participants were adult women at risk of pregnancy (18-50 years of age) living in Oregon from January 1, 2012, through December 31, 2017, and enrolled in Medicaid (532 337 person-years) or in commercial health insurance (1 131 738 person-years).

**Exposure:**

Implementation of an effective contraceptive use incentive metric as defined using the 2019 Oregon Health Authority specifications.

**Main Outcomes and Measures:**

*International Classification of Diseases, Ninth Revision* codes; *International Statistical Classification of Diseases and Related Health Problems, Tenth Revision* codes; and *Current Procedural Terminology* codes were used to identify contraceptive use. Annual rates of effective contraceptive use were measured through health insurance claims.

**Results:**

The final analyses included 532 337 Medicaid person-years and 1 131 738 privately insured person-years. Women enrolled in Medicaid were younger than those with private insurance (47.5% vs 33.2% of women in 2013 younger than 30 years), and approximately 40% of Medicaid enrollees (vs fewer than 10% of women with private insurance) resided in rural locations. Demographic characteristics within each group remained similar before and after the incentive metric was implemented. In the comparative interrupted time series model, relative to the commercially insured comparison group, effective contraceptive use among Medicaid enrollees for all ages combined increased 3.6% (95% CI, 3.1%-4.1%) 1 year after the start of the incentive metric, 7.5% (95% CI, 6.8%-8.2%) at the end of 2 years, and 11.5% (95% CI, 10.5%-12.4%) at the end of 3 years. Prior to the introduction of the incentive, contraceptive use rates among the youngest cohort of Medicaid enrollees (18-24 years of age) were decreasing; following the introduction of the incentive, contraceptive use increased steadily among all enrollees. Among women aged 18 to 24 years, the effective contraceptive use rate increased 16.5 percentage points (95% CI, 14.4-18.6 percentage points) after 3 years. The largest initial increase in contraceptive use was among women enrolled in Medicaid who were 30 to 34 years of age (4.9%; 95% CI, 3.4%-6.3%).

**Conclusions and Relevance:**

Implementation of the effective contraceptive use incentive metric was associated with a significant increase in contraceptive use among Medicaid enrollees relative to a commercially insured comparison group. This finding is relevant given national efforts aimed at adopting a similar metric for widespread use.

## Key Points

**Question:**

Does an association exist between a program-level incentive metric for contraceptive use among women enrolled in Medicaid and the increased use of effective contraceptive methods?

**Findings:**

This cohort study using a comparative interrupted time series analysis of 532 337 Medicaid person-years and 1 131 738 privately insured person-years found that a program level incentive was associated with an increase in the use of effective contraception among women enrolled in the Oregon Medicaid program relative to changes in contraceptive use by commercially insured women.

**Meaning:**

The findings of this study support program-based incentives for the provision of effective contraception use among women enrolled in Medicaid.

## Teaser

This statewide cohort study of more than 1.5 million person-years uses a comparative interrupted time series analysis to assess whether implementing an incentive metric is associated with effective contraceptive use among women enrolled in the Oregon Medicaid program.

## Introduction

Unintended pregnancies are endemic in the United States, accounting nearly half of all pregnancies.^1^ The personal, social, and economic costs and the health consequences associated with unintended pregnancies are considerable. The cost of unintended pregnancy for the Medicaid program—which provides coverage for a large proportion of women with low income who are of reproductive age—is substantial.^2,3,4^ Effective strategies to improve the use of voluntary contraception can be beneficial to individuals while also reducing the cost to the Medicaid program.

Since the passage of the Affordable Care Act, states have sought innovative methods to achieve the triple aim of health care: to improve population health outcomes and patient experience of care while reducing costs.^5,6^ Financial incentives and quality metrics are increasingly common in Medicaid and across the health system for a range of health outcomes, including effective contraceptive use (ECU).^7,8,9^ However, limited data exist on the associations between incentivizing ECU, contraceptive behavior, and health outcomes.

In January 2015, Oregon became the first state to implement a financial incentive for providing effective contraception within their Medicaid program. The Oregon ECU metric assesses the percentage of reproductive age women at risk for unintended pregnancy who are provided with an effective contraceptive method.^10^ The Oregon Health Authority defines ECU as sterilization, intrauterine device, progestin injectable, contraceptive implant, pill, patch, ring, and diaphragm.

The ECU metric is one of 17 quality metrics included in a bonus pool for the Oregon coordinated care organizations (CCOs), a delivery system model that provides care for more than 90% of the state’s Medicaid beneficiaries.^11,12,13^ The incentives are significant: the CCOs that meet quality metrics are eligible for a bonus equal to 4% of their total Medicaid budget.^14^ Lessons learned from Oregon have national relevance. A similar metric, developed by the federal Office of Population Affairs, has since been endorsed by the National Quality Forum as a quality indicator for national use.^15^ However, it has not yet been implemented nationally.

Incentive measure benchmarks are selected by the state and are meant to be aspirational goals. The CCOs are not expected to meet the benchmark each year but rather to make improvement toward the benchmark. The state benchmark for the ECU metric is 50% of all reproductive age women at risk for pregnancy using an effective method of contraception, or an improvement in the score from the prior year. Improvement targets are based on the Minnesota Department of Health Quality Incentive Payment System, which requires at least 10% reduction in the gap between baseline and the benchmark to qualify for incentive payments.^10^

The state recommends that CCOs increase voluntary contraceptive use through the following strategies: screen women for their pregnancy intention annually; remove access barriers to contraception; enhance partnerships with Title X family planning clinics; improve availability and uptake of long-acting, reversible contraception; create quality improvement processes; and offer clinician training. However, it is ultimately the decision of the CCO as to which strategies they implement to increase voluntary contraceptive use. In the present study, we seek to understand how financial incentives may be associated with ECU, by using claims data from the state of Oregon to compare changes among women enrolled in Medicaid with those having commercial insurance.

## Methods

We conducted a secondary analysis of claims data and used a quasi-experimental design to assess the association of the ECU metric incentive with ECU rates in Oregon. All analyses and reporting of results were conducted in accordance with the Strengthening the Reporting of Observational Studies in Epidemiology (STROBE) reporting guideline for cohort studies.^16^ The institutional review board at Oregon Health & Science University reviewed and approved this study and waived the requirement for obtaining participant informed consent because all data were deidentified. No one received compensation or was offered any incentive for participating in this study.

### Study Population and Data

We used claims and enrollment information for all adult women at risk of pregnancy living in Oregon from January 1, 2012, through December 31, 2017. All outcomes and patient characteristics were measured at the patient-year level and included enrollment and claims information for the calendar year (January 1 through December 31). Women at risk of pregnancy included adult women 18 to 50 years of age and excluded women who were unable to become pregnant due to a history of hysterectomy, menopause, bilateral oophorectomy, or congenital abnormality. The incentive metric did not apply to adolescents.

Incentive metrics were implemented only within the Medicaid program. No similar initiative occurred within commercially insured plans. To examine the association of the incentive metrics, our data included both claims and enrollment information for Medicaid beneficiaries (intervention group) and commercially insured individuals (comparison group).^17^ We obtained Medicaid data from the Oregon Health Authority. We received data on the commercially insured women from the Oregon All Payer All Claims (APAC) Reporting Program. The APAC database contains claims from the largest commercial insurers in Oregon, excluding information from self-insured plans, small carriers (<5000 enrollees), and the Federal Employee Health Benefits Program; the APAC program covers an estimated 87% of privately insured Oregonians.^17,18^

To examine the association of the intervention with the Medicaid population, we excluded from our sample beneficiaries who were enrolled in their insurance plan for less than 11 months in a year, beneficiaries who were enrolled in both Medicaid and Medicare (dual eligibility), and beneficiaries who were enrolled in Medicaid but not enrolled with a CCO. We also excluded individuals who were newly eligible for Medicaid as part of the 2014 expansion of Medicaid eligibility under the Affordable Care Act because this population is likely to differ from the traditional Medicaid population in unobservable ways that may bias our estimates of contraception use. The full set of selection criteria, along with sample sizes, is available in the eFigure in the Supplement.

### Variables

Our primary outcome was the annual rate of effective contraceptive use during each calendar year.^19^ We used the Oregon Health Authority 2019 specifications for the incentive metric to calculate this measure.^19^ *International Classification of Diseases, Ninth Revision* codes; *International Statistical Classification of Diseases and Related Health Problems, Tenth Revision* codes; and *Current Procedural Terminology* codes were used to identify contraceptive use in our database.

In brief, the numerator of the metric consisted of individuals using sterilization, intrauterine device, implant, injection, hormonal contraception, or diaphragm. Female sterilization was considered a permanent numerator qualifier: women with any history of sterilization were considered current contraception users. The denominator included women at risk of pregnancy, which was defined as women aged 18 to 50 years. Women were excluded from the denominator if they had any record of hysterectomy, surgical or natural menopause, or infertility. The full technical specifications for the metric, including the *International Classification of Diseases* and *Current Procedural Terminology* codes, have been published by the Oregon Health Authority.^19^

Model covariates included age group (18-24, 25-29, 30-34, and 35-50 years), rural vs urban residence, type of insurance coverage (Medicaid or commercial), and health status.^20^ We adjusted for health status using a modified version of the Chronic Illness and Disability Payment System (CDPS), a claims-based risk adjustment model.^21^ Each binary CDPS indicator represented whether a woman had claims for the particular condition during the year. We chose to exclude CDPS indicators for pregnancy from our models because pregnancy is potentially downstream from our outcome. We also excluded indicators for substance use, HIV or AIDS, and infection because these conditions were censured in commercial insurance claims.

### Statistical Analysis

We tested our hypothesis using a comparative interrupted time series (CITS) approach modeled at the patient-year level. Similar to a standard ITS approach, the CITS approach compares before vs after intervention outcomes after accounting for time trends.^22,23,24^ Unlike for a standard ITS design, we included the Oregon commercially insured population as a comparison group, which enabled us to control for systemwide changes that may have occurred near the time of the intervention.

We modeled yearly ECU for all women, and also for each age group (18-24, 25-29, 30-34, and 35-50 years) separately using a linear probability model with the following specification: *Y_it_* = β_0_ + β_1_ × Medicaid*_i_* + β_2_ × Medicaid*_i_* × Time*_t_* + β_3_ × Medicaid*_i_* × Intervention*_t_* + β_4_ × Medicaid*_i_* × Time*_t_* × Intervention*_t_* + ∑ (β_year_ × Year*_t_*) + β*_x_* × *X_it_*.

Our outcome for all models was a binary indicator of contraception use for woman *i* in year *t*. Our models included a Medicaid by time interaction (β_2_) to account for baseline (β_0_) differences in trend by payer, a Medicaid by intervention interaction (β_3_) to measure the discrete change after the policy, and a Medicaid by time by intervention interaction (β_4_) to measure changes in the time trend following the policy. Models also included a main effect for Medicaid (β_1_) to measure baseline differences between payers, and year fixed effects (β_year_) to measure noncontinuous systemwide changes during each Year*_t_*. Although we used a categorical measure of years (Year*_t_*) to estimate the systemwide changes to the ECU rate, we assumed that the effect of time on each payer differed linearly as a function of a continuous measure of years (Time*_t_*). All models were adjusted for patient indicators for CDPS disease groups and for rurality (*X_it_*). The patient indicators for the all-ages model included a term adjusting for age group. For each model, we estimated the effect associated with the policy at 1, 2, and 3 years after the intervention, using a linear combination of β_3_ and β_4_. Because individual patients might contribute more than 1 year of person-time, standard errors were adjusted for clustering at the individual level by using a Huber-White sandwich estimator. This estimator also has the added benefit of correcting for nonnormally distributed errors that occur when running a linear probability model.

We visually assessed the difference between 2 different sets of estimated ECU rates: (1) estimated ECU rates for the Medicaid population that included the effect associated with the policy and (2) estimated ECU rates for the Medicaid population with the effect associated with the policy removed. Both sets are calculated by applying the coefficients from the CITS models to the study population at each year. The postpolicy period estimations without the effect associated with the policy are estimated using a combination of prepolicy time trends and observed changes in the comparison group (commercially insured women). This value also includes the estimated rates among the commercial population for reference.

We conducted 2 sensitivity analyses to test whether our results were robust to different model specifications. First, we ran models that allowed the effect associated with the policy to vary nonlinearly over time. Second, we ran all models, excluding the commercially insured comparison group, equivalent to a standard ITS approach.^25^ We do not present results for a difference-in-difference model because the preintervention trends differed for commercially insured women and Medicaid enrolled women.^26,27^

All hypothesis tests were 2-sided, and we considered *P* < .05 to be statistically significant. Data management and analyses were conducted using R, version 3.6.0 (R Project for Statistical Computing).

## Results

Our analyses included 532 337 Medicaid person-years and 1 131 738 privately insured person-years. We selected 1 year in the preintervention period (2013) and 1 year in the postintervention period (2016) to descriptively assess changes to demographic characteristics over time. The number of women enrolled in Medicaid increased from 2013 to 2016 (69 076 before intervention to 95 526 after intervention), while the number of women enrolled in private insurance stayed relatively stable (from 193 873 to 183 131). Consistent with previous findings, key demographic characteristic differences existed between women enrolled in Medicaid vs private insurance.^28^ Women enrolled in Medicaid were younger, with 47.5% of women in 2013 younger than 30 years (vs 33.2% with private insurance). Approximately 40% of Medicaid enrollees resided in rural locations, as compared with less than 10% of women with private insurance. Demographic characteristics within each group remained consistent before and after the policy change incentivizing contraceptive use was implemented, with one exception. Women enrolled in Medicaid tended to have more chronic conditions than women enrolled in private insurance, but this gap shrank in the postintervention period (Table 1).

**Table 1.** Demographic Information for Women at Risk of Pregnancy Before and After Policy Implementation, by Payer Type^a^

Characteristic	Participants with Medicaid insurance, No. (%)		Participants with private insurance, No. (%)	
	Preintervention (n = 69 076)	Postintervention (n = 95 526)	Preintervention (n = 193 873)	Postintervention (n = 183 131)
Age group, y				
18-24	20 655 (29.9)	29 618 (31.0)	41 182 (21.2)	38 466 (21.0)
25-29	12 125 (17.6)	17 324 (18.1)	23 314 (12.0)	24 470 (13.4)
30-34	11 974 (17.3)	16 410 (17.2)	28 261 (14.6)	27 507 (15.0)
35-50	24 322 (35.2)	32 174 (33.7)	101 116 (52.2)	92 688 (50.6)
Geographic area				
Rural	27 385 (39.6)	37 886 (39.7)	16 272 (8.4)	15 090 (8.2)
Urban	41 691 (60.4)	57 640 (60.3)	177 601 (91.6)	168 041 (91.8)
Effective contraception use^b^	27 150 (39.3)	42 344 (44.3)	60 569 (31.2)	61 306 (33.5)
CDPS risk score, mean (SD)^c^	1.14 (0.99)	1.02 (0.84)	0.79 (0.58)	0.79 (0.58)

Abbreviation: CDPS, Chronic Illness and Disability Payment System.

^a^ Before policy estimates were for women enrolled in 2013, whereas after policy estimates were from women enrolled in 2016.

^b^ Measured and calculated per the Oregon Health Authority 2019 specification for the effective contraceptive use metric.

^c^ Calculated using a modified CDPS risk score that excludes weights for pregnancy, substance use, HIV or AIDS, and infection.

We examined the observed rates of ECU for each age cohort by insurer type before and after the policy change and contrasted this with the estimated rate of contraceptive use among Medicaid beneficiaries as though the incentive metric were not introduced (Figure). Immediately following the policy intervention, the estimated and observed rates of contraception use quickly diverged, suggesting that the incentive metric was associated with the overall trajectory of contraceptive use by Medicaid enrollees. By contrast, trends in contraceptive use were generally consistent among commercially insured women, with no inflection point following the implementation of the Medicaid policy. These findings were consistent across age cohorts.

**Figure.**
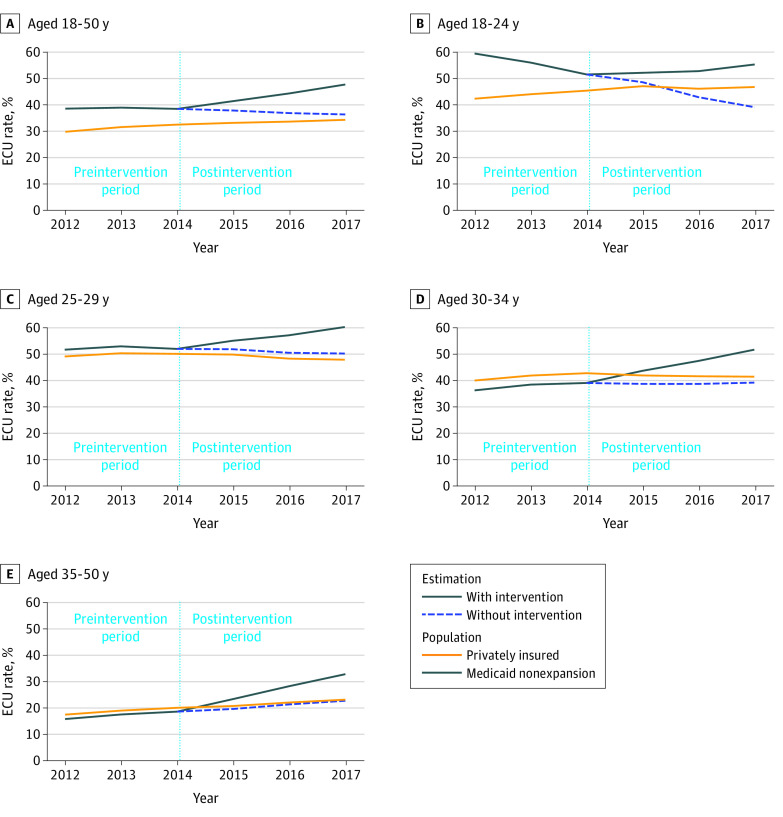
Estimated Effective Contraception Use (ECU) Rates Over Time Before and After Policy Implementation, by Age Group

Table 2 gives the results of our statistical analyses for the change in ECU among Medicaid enrollees. Relative to the commercially insured comparison group, ECU increased 3.6% (95% CI, 3.1%-4.1%) 1 year after the policy change that implemented the incentive metric, 7.5% (95% CI, 6.8%-8.2%) after 2 years, and 11.5% (95% CI, 10.5%-12.4%) after 3 years. Although the largest initial increase in contraceptive use was for women 30 to 34 years of age (4.9%; 95% CI, 3.4%-6.3%), the youngest cohort of women (18-24 years of age) appeared to have experienced the greatest benefit. Prior to the introduction of the incentive, contraceptive use rates among the youngest cohort of Medicaid enrollees were decreasing; following the introduction of the incentive, contraceptive use increased steadily among all enrollees. Among women aged 18 to 24 years, the ECU rate increased 16.5 percentage points (95% CI, 14.4-18.6 percentage points) after 3 years (Table 2). Each age cohort, when examined separately, experienced an annual increase in ECU following the policy change.

**Table 2.** Change in Effective Contraception Use in Medicaid Beneficiaries Relative to the Commercially Insured Comparison Group^a^

Age group, y	Relative change, % (95% CI)		
	Year 1 (2015) estimate	Year 2 (2016) estimate	Year 3 (2017) estimate
All ages (18-50)	3.6 (3.1-4.1)	7.5 (6.8-8.2)	11.5 (10.5-12.4)
18-24	3.6 (2.5-4.8)	10.0 (8.5-11.6)	16.5 (14.4-18.6)
25-29	3.0 (1.4-4.6)	6.2 (4.1-8.3)	9.4 (6.5-12.3)
30-34	4.9 (3.4-6.3)	8.6 (6.6-10.5)	12.3 (9.6-15.0)
35-50	3.7 (3.0-4.4)	6.8 (5.9-7.7)	9.9 (8.6-11.2)

^a^ All *P* < .001.

Our results were robust to a variety of sensitivity analyses. Models that allowed for nonlinear policy changes produced results that were qualitatively identical to those in the standard model (eTable 1 in the Supplement). Standard ITS models, including only within the Medicaid population, also produced similar results (eTable 2 in the Supplement). The results of the full model output from the CITS models for each age group used to populate Table 2 are presented in eTables 3, 4, 5, 6, and 7 in the Supplement.

## Discussion

In this statewide study of a policy change implementing an incentive metric for contraceptive use among Medicaid enrollees, we observed a substantial change in the trend of contraceptive use for Medicaid enrollees relative to the privately insured. A consistent annual increase in contraceptive use among Medicaid enrollees was observed among all age groups after the policy change. We found that implementation of the incentive metric was associated with a reversal of declining contraceptive use among young women. Prior to the introduction of the incentive, contraceptive use rates among the youngest cohort of women (18-24 years of age) were decreasing; following the introduction of the incentive, the trend reversed, and these rates began to increase.

One strength of this study is the use of data covering most Medicaid-insured and commercially insured patients in a single state. Those data allowed for comparisons across insurance types and over time. Our use of the quasi-experimental CITS design showed a marked increase in contraceptive use in the Medicaid population above what would have been expected from baseline trends. No similar increase was seen in our comparison group, privately insured women.

In considering incentive metrics and reproductive health, it is paramount to acknowledge the reality of reproductive coercion in the US and globally.^29^ In developing this metric, the standards and principles of a human rights–based approach to family planning were carefully considered.^30^ The financial incentive to provide effective contraception is applied at the CCO level, not at the level of the individual clinician or clinic. This ensures that the health care professional does not have a vested financial interest in a woman’s contraceptive use. Similarly, the benchmark for success with this metric is 50% or an improvement from the previous year’s score. This threshold was set with the recognition that many women will desire pregnancy, and it is equally important to help them achieve a healthy pregnancy and birth.

### Limitations

Our results should be interpreted with the following limitations in mind. Administrative claims data are not collected specifically for research purposes, and as such, we have limited demographic information. Our data set does not contain information on women’s reproductive goals, sexual activity, or parity, all of which would influence contraceptive use. We also lack data on what interventions were put into place by CCOs to achieve this metric. Similarly, owing to a lack of specificity in the *International Classification of Diseases, Ninth Revision* codes, we are able to assess ECU only overall and were unable to distinguish between initiation or continuation of all method types for women in our sample. This is a key distinction for understanding any potential association with unintended pregnancy: in 2008, the one-third of women at risk for unintended pregnancy not using any contraception accounted for 95% of all unintended pregnancies.^31^ Those at greatest risk for unintended pregnancy are the population we found benefited the most from the ECU metric: young women.^32^ The baseline trends in contraceptive use in our Medicaid population and comparison group, the privately insured, were not parallel. The trends in baseline were most markedly different among the youngest cohort of women. Although our analytic approach, CITS, does not require that the baseline trends be parallel, this difference in trends could mean that the 2 groups did not respond similarly to systemwide changes. This could lead to biases in our estimates, especially if the comparison group underwent large changes that would not have affected the Medicaid population. This scenario is unlikely, however, because models excluding the comparison group produced similar results. Finally, our data are from 1 state, Oregon, with a relatively homogeneous Medicaid population. This affects generalizability for other states. In particular, Oregon’s metric included use of a diaphragm as an effective method of contraception. Although this inclusion deviates from the Centers for Disease Control and Prevention definition of ECU, it is in line with the definition of the Office of Population Affairs as a moderately effective method.

## Conclusions

Supporting individuals in achieving their reproductive goals through access to voluntary family planning is a national and international public health goal. Our results support a role of incentive metrics to strengthen ECU among women at risk of unintended pregnancy in a state Medicaid program. Our findings have national relevance: although the Office of Population Affairs and the National Quality Forum have endorsed a similar metric for use nationally, these metrics have not been broadly implemented by state Medicaid programs or by commercial insurers. States seeking to monitor access to effective contraception, increase voluntary contraceptive use, and decrease unintended pregnancy may want to consider similar incentive programs using a claims-based metric.

## Notes

Supplement. **eTable 1.** Estimated Percentage Point Change in Effective Contraception Rates Associated With the Policy by Year From Model Allowing for a Non-Linear Policy Effect **eTable 2.** Estimated Percentage Point Change in Effective Contraception Rates Associated With the Policy by Year From Model Without Control Group (Standard ITS) **eTable 3.** Raw Model Output From Primary Comparative Interrupted Time Series Model for Women of All Ages (18-50) **eTable 4.** Raw Model Output From Primary Comparative Interrupted Time Series Model for Women Ages 18-24 **eTable 5.** Raw Model Output From Primary Comparative Interrupted Time Series Model for Women Ages 25-29 **eTable 6.** Raw Model Output From Primary Comparative Interrupted Time Series Model for Women Ages 30-34 **eTable 7.** Raw Model Output From Primary Comparative Interrupted Time Series Model for Women Ages 35-50 **eFigure.** CONSORT Diagram of Sample Selection

## References

[1] Finer LB, Zolna MR Declines in unintended pregnancy in the United States, 2008-2011. N Engl J Med. 2016;374(9):843-852. doi:10.1056/NEJMsa1506575 26962904 PMC4861155

[2] Renfro S, Lindner S, McConnell KJ Decomposing Medicaid spending during health system reform and ACA expansion: evidence from Oregon. Med Care. 2018;56(7):589-595. doi:10.1097/MLR.0000000000000928 29762274

[3] Sonfield A, Kost K, Gold RB, Finer LB The public costs of births resulting from unintended pregnancies: national and state-level estimates. Perspect Sex Reprod Health. 2011;43(2):94-102. doi:10.1363/4309411 21651708

[4] Sonfield A, Kost K. Public Costs From Unintended Pregnancies and the Role of Public Insurance Programs in Paying for Pregnancy and Infant Care: Estimates for 2008. Guttmacher Institute; 2013.

[5] Berwick DM, Nolan TW, Whittington J The triple aim: care, health, and cost. Health Aff (Millwood). 2008;27(3):759-769. doi:10.1377/hlthaff.27.3.759 18474969

[6] Gusmano MK, Thompson FJ An examination of Medicaid delivery system reform incentive payment initiatives under way in six states. Health Aff (Millwood). 2015;34(7):1162-1169. doi:10.1377/hlthaff.2015.0165 26153311

[7] Maternal and Infant Health Initiative Contraceptive Care Measures Measure CCW. Published 2017 Accessed April 22, 2019. https://www.medicaid.gov/medicaid/quality-of-care/improvement-initiatives/maternal-infant-health-care-quality/index.html

[8] Blumenthal D, McGinnis JM Measuring vital signs: an IOM report on core metrics for health and health care progress. JAMA. 2015;313(19):1901-1902. doi:10.1001/jama.2015.4862 25919301

[9] Sonfield A Pay-for-performance: making it work for safety-net family planning centers and the clients they serve. Guttmacher Policy Rev. 2014;17(2):8-13.

[10] Oregon Health Authority. Effective contraceptive use among women at risk of unintended pregnancy guidance document. Published December 22, 2014. Accessed June 22, 2020. https://www.oregon.gov/oha/HPA/ANALYTICS/CCOMetrics/2014-Effective-Contraceptive-Use-Guidance-Document.pdf

[11] McConnell KJ Oregon’s Medicaid coordinated care organizations. JAMA. 2016;315(9):869-870. doi:10.1001/jama.2016.0206 26847402 PMC4939819

[12] McConnell KJ, Chang AM, Cohen DJ, Oregon’s Medicaid transformation: an innovative approach to holding a health system accountable for spending growth. Healthc (Amst). 2014;2(3):163-167. doi:10.1016/j.hjdsi.2013.11.002 25540719 PMC4273859

[13] McConnell KJ, Renfro S, Chan BK, Early performance in Medicaid Accountable Care Organizations: a comparison of Oregon and Colorado. JAMA Intern Med. 2017;177(4):538-545. doi:10.1001/jamainternmed.2016.9098 28192568 PMC5440252

[14] Oregon Health Authority; Office of Health Analytics. CCO metrics. Accessed July 6, 2020. https://www.oregon.gov/oha/hpa/analytics/pages/cco-metrics.aspx

[15] Office of Population Affairs Clinical performance measures of contraceptive care. Published 2018. Accessed April 22, 2019. https://www.hhs.gov/opa/sites/default/files/clinical-performance-measures.pdf

[16] von Elm E, Altman DG, Egger M, Pocock SJ, Gøtzsche PC, Vandenbroucke JP; STROBE Initiative The Strengthening the Reporting of Observational Studies in Epidemiology (STROBE) statement: guidelines for reporting observational studies. J Clin Epidemiol. 2008;61(4):344-349. doi:10.1016/j.jclinepi.2007.11.008 18313558

[17] Oregon Health Authority. Oregon All Payer All Claims Reporting Database (APAC): an overview. Published March 2018. Accessed October 1, 2019. https://www.oregon.gov/oha/HPA/ANALYTICS/APAC%20Page%20Docs/APAC-Overview.pdf

[18] Bland SE, Crowley JS, Gostin LO Strategies for health system innovation after Gobeille v Liberty Mutual Insurance Co. JAMA. 2016;316(6):581-582. doi:10.1001/jama.2016.8293 27367856

[19] Oregon Health Authority. Effective contraceptive use. Updated February 15, 2019. Accessed June 22, 2020. https://www.oregon.gov/oha/HPA/ANALYTICS/CCOMetrics/2019-Effective-Contraceptive-Use.pdf

[20] US Department of Agriculture: Ag Data Commons. Rural-urban commuting area codes. Accessed March 1, 2019. https://data.nal.usda.gov/dataset/rural-urban-commuting-area-codes

[21] Kronick R, Gilmer T, Dreyfus T, Lee L Improving health-based payment for Medicaid beneficiaries: CDPS. Health Care Financ Rev. 2000;21(3):29-64. 11481767 PMC4194678

[22] Lopez Bernal J, Cummins S, Gasparrini A The use of controls in interrupted time series studies of public health interventions. Int J Epidemiol. 2018;47(6):2082-2093. doi:10.1093/ije/dyy135 29982445

[23] MDCR. Working paper on research methodology: the validity and precision of the comparative interrupted time series design and the difference-in-difference design in educational evaluation. Published September 2013. Accessed June 22, 2020. https://www.mdrc.org/sites/default/files/validity_precision_comparative_interrupted_time_series_design.pdf

[24] St.Clair T, Hallberg K, Cook T The validity and precision of the comparative interrupted time-series design. J Educ Behav Stat. 2016;41:269-299. doi:10.3102/1076998616636854

[25] Bernal JL, Cummins S, Gasparrini A Interrupted time series regression for the evaluation of public health interventions: a tutorial. Int J Epidemiol. 2017;46(1):348-355. 27283160 10.1093/ije/dyw098 PMC5407170

[26] Pishke A Mostly Harmless Econometrics. Princeton University Press; 2009.

[27] Ryan AM, Burgess JF Jr, Dimick JB Why we should not be indifferent to specification choices for difference-in-differences. Health Serv Res. 2015;50(4):1211-1235. doi:10.1111/1475-6773.12270 25495529 PMC4545355

[28] Kaiser Family Foundation. Medicaid’s role for women. Published March 2019. Accessed June 22, 2020. http://files.kff.org/attachment/Fact-Sheet-Medicaids-Role-for-Women

[29] American College of Obstetricians and Gynecologists ACOG Committee opinion no. 554: reproductive and sexual coercion. Obstet Gynecol. 2013;121(2, pt 1):411-415. doi:10.1097/01.AOG.0000426427.79586.3b 23344307

[30] World Health Organization. Ensuring human rights in the provision of contraceptive information and services: guidance and recommendations. Published 2014. Accessed June 22, 2020. https://apps.who.int/iris/bitstream/handle/10665/102539/9789241506748_eng.pdf;jsessionid=59CA87902FB72098FE90EA1FD2DFB435?sequence=1 24696891

[31] Guttmacher Institute. Contraceptive use in the United States. Published April 2020. Accessed June 22, 2020. https://www.guttmacher.org/fact-sheet/contraceptive-use-united-states

[32] Jones J, Mosher W, Daniels K Current contraceptive use in the United States, 2006–2010, and changes in patterns of use since 1995. Natl Health Stat Report. 2012;(60):1-25. 24988814

