# Supplementary material for: Association of Implementing an Incentive Metric in the Oregon Medicaid Program With Effective Contraceptive Use
Source: JAMA Netw Open. 2020 Aug 5;3(8):e2012540. doi: 10.1001/jamanetworkopen.2020.12540 (PMC7407076; doi:10.1001/jamanetworkopen.2020.12540)
Supplement: Supplement. — eTable 1. Estimated Percentage Point Change in Effective Contraception Rates Associated With the Policy by Year From Model Allowing for a Non-Linear Policy Effect eTable 2. Estimated Percentage Point Change in Effective Contraception Rates Associated With the Policy by Year From Model Without Control Group (Standard ITS) eTable 3. Raw Model Output From Primary Comparative Interrupted Time Series Model for Women of All Ages (18-50) eTable 4. Raw Model Output From Primary Comparative Interrupted Time Series Model for Women Ages 18-24 eTable 5. Raw Model Output From Primary Comparative Interrupted Time Series Model for Women Ages 25-29 eTable 6. Raw Model Output From Primary Comparative Interrupted Time Series Model for Women Ages 30-34 eTable 7. Raw Model Output From Primary Comparative Interrupted Time Series Model for Women Ages 35-50 eFigure. CONSORT Diagram of Sample Selection [file jamanetwopen-3-e2012540-s001.pdf]

Supplementary Online Content

Rodriguez MI, Meath T, Huang J, Darney BG, McConnell KJ. Association of implementing an incentive metric in the Oregon Medicaid program with effective contraceptive use. *JAMA Netw Open*. 2020;3(8):e2012540. doi:10.1001/jamanetworkopen.2020.12540

eTable 1. Estimated Percentage Point Change in Effective Contraception Rates Associated With the Policy by Year From Model Allowing for a Non-Linear Policy Effect

eTable 2. Estimated Percentage Point Change in Effective Contraception Rates Associated With the Policy by Year From Model Without Control Group (Standard ITS)

eTable 3. Raw Model Output From Primary Comparative Interrupted Time Series Model for Women of All Ages (18-50)

eTable 4. Raw Model Output From Primary Comparative Interrupted Time Series Model for Women Ages 18-24

eTable 5. Raw Model Output From Primary Comparative Interrupted Time Series Model for Women Ages 25-29

eTable 6. Raw Model Output From Primary Comparative Interrupted Time Series Model for Women Ages 30-34

eTable 7. Raw Model Output From Primary Comparative Interrupted Time Series Model for Women Ages 35-50

eFigure. CONSORT Diagram of Sample Selection

This supplementary material has been provided by the authors to give readers additional information about their work.

© 2020 Rodriguez MI et al. *JAMA Network Open*.

eTable 1. Estimated Percentage Point Change in Effective Contraception Rates Associated With the Policy by Year From Model Allowing for a Non-Linear Policy Effect

		Policy effect by year							
		Year 2015			Year 2016			Year 2017	
		Estimate (95% CI)	p-value		Estimates (95%)	P-value		Estimates (95%)	P-value
**Age Group**									
**All Ages (18-50)**		3.6% (0.031, 0.041)	< 0.01		7.6% (0.068, 0.083)	< 0.01		11.4% (0.105, 0.124)	< 0.01
**18-24**		3.7% (0.026, 0.048)	< 0.01		9.9% (0.083, 0.115)	< 0.01		16.5% (0.144, 0.186)	< 0.01
**25-29**		2.9% (0.013, 0.045)	< 0.01		6.5% (0.042, 0.087)	< 0.01		9.3% (0.064, 0.122)	< 0.01
**30-34**		4.8% (0.033, 0.063)	< 0.01		8.7% (0.067, 0.108)	< 0.01		12.2% (0.095, 0.149)	< 0.01
**35-50**		3.5% (0.028, 0.042)	< 0.01		7.1% (0.061, 0.081)	< 0.01		9.7% (0.084, 0.110)	< 0.01

© 2020 Rodriguez MI et al. *JAMA Network Open*.

eTable 2. Estimated Percentage Point Change in Effective Contraception Rates Associated With the Policy by Year From Model Without Control Group (Standard ITS)

		Policy effect by year							
		Year 2015			Year 2016			Year 2017	
		Estimate (95% CI)	p-value		Estimates (95%)	P-value		Estimates (95%)	P-value
**Age Group**									
**All Ages (18-50)**		2.9% (0.025, 0.033)	< 0.01		6.1% (0.055, 0.067)	< 0.01		9.2% (0.084, 0.100)	< 0.01
**18-24**		3.7% (0.028, 0.046)	< 0.01		8.6% (0.074, 0.098)	< 0.01		13.5% (0.118, 0.151)	< 0.01
**25-29**		2.1% (0.009, 0.032)	< 0.01		4.0% (0.024, 0.056)	< 0.01		5.9% (0.037, 0.081)	< 0.01
**30-34**		2.5% (0.014, 0.037)	< 0.01		4.7% (0.031, 0.062)	< 0.01		6.8% (0.047, 0.089)	< 0.01
**35-50**		3.1% (0.025, 0.037)	< 0.01		6.1% (0.053, 0.069)	< 0.01		9.1% (0.080, 0.103)	< 0.01

© 2020 Rodriguez MI et al. *JAMA Network Open*.

eTable 3. Raw Model Output From Primary Comparative Interrupted Time Series Model for Women of All Ages (18-50)

	Estimate	Std. Error	t value	Pr(>|t|)
(Intercept)	0.431491	0.001402	307.6979	0
medicaid	0.027658	0.002916	9.484393	2.44E-21
year2	0.015589	0.000965	16.15591	1.03E-58
year3	0.023528	0.001206	19.50187	1.06E-84
year4	0.02932	0.00127	23.09448	5.3E-118
year5	0.031139	0.00127	24.52805	7.4E-133
year6	0.037056	0.001329	27.88063	4.6E-171
rural	0.002389	0.001435	1.664855	0.095942
car	0.005539	0.001702	3.255262	0.001133
psy	0.067605	0.001176	57.51074	0
skc	0.011144	0.001481	7.524731	5.28E-14
cns	-0.02082	0.002555	-8.14951	3.65E-16
pul	0.026716	0.001631	16.37632	2.82E-60
gi	0.032852	0.00161	20.40639	1.47E-92
dia	-0.03303	0.002828	-11.6793	1.63E-31
skn	0.011711	0.001979	5.917894	3.26E-09
ren	0.001935	0.003229	0.59909	0.549113
can	-0.04105	0.003941	-10.4174	2.06E-25
dd	0.035293	0.011928	2.958811	0.003088
gen	0.195374	0.001963	99.54504	0
met	-0.00714	0.002697	-2.64852	0.008085
eye	-0.01835	0.004842	-3.78982	0.000151
cer	-0.05914	0.009934	-5.9531	2.63E-09
hem	0.055826	0.004397	12.69733	6.12E-37
agegroup25-29	0.020327	0.001607	12.64577	1.18E-36
agegroup30-34	-0.07184	0.00169	-42.4983	0
agegroup35+	-0.27572	0.001349	-204.329	0

© 2020 Rodriguez MI et al. *JAMA Network Open*.

medicaid:time	-0.01172	0.001169	-10.0222	1.22E-23
medicaid:intervention	-0.12114	0.00655	-18.495	2.27E-76
medicaid:time:intervention	0.039291	0.001709	22.98549	6.5E-117

© 2020 Rodriguez MI et al. *JAMA Network Open*.

eTable 4. Raw Model Output From Primary Comparative Interrupted Time Series Model for Women Ages 18-24

	Estimate	Std. Error	t value	Pr(>|t|)
(Intercept)	0.384957	0.002437	157.9316	0
medicaid	0.175797	0.006188	28.40717	1.6E-177
year2	0.016759	0.002382	7.034701	2E-12
year3	0.0281	0.003095	9.079124	1.09E-19
year4	0.044975	0.003247	13.85255	1.23E-43
year5	0.038168	0.003186	11.97927	4.56E-33
year6	0.042798	0.003394	12.61175	1.82E-36
rural	0.030409	0.002688	11.31443	1.11E-29
car	0.050475	0.005468	9.230954	2.68E-20
psy	0.130022	0.002435	53.38893	0
skc	0.048035	0.003656	13.13861	1.98E-39
cns	-0.02496	0.006873	-3.63081	0.000283
pul	0.079516	0.003524	22.56585	9.4E-113
gi	0.087392	0.003954	22.10086	3.1E-108
dia	-0.01489	0.010196	-1.46066	0.144108
skn	0.06014	0.004348	13.83272	1.62E-43
ren	0.017609	0.009761	1.80401	0.07123
can	-0.017	0.018832	-0.90259	0.366744
dd	-0.05736	0.017116	-3.35139	0.000804
gen	0.217151	0.003994	54.36545	0
met	-0.00181	0.006893	-0.26219	0.793173
eye	0.001118	0.016645	0.067164	0.946451
cer	-0.09259	0.034866	-2.65555	0.007918
hem	0.091849	0.010394	8.836915	9.84E-19
medicaid:time	-0.04768	0.002559	-18.6342	1.7E-77
medicaid:intervention	-0.2204	0.014029	-15.7107	1.28E-55
medicaid:time:intervention	0.064166	0.003698	17.34932	2E-67

© 2020 Rodriguez MI et al. *JAMA Network Open*.

eTable 5. Raw Model Output From Primary Comparative Interrupted Time Series Model for Women Ages 25-29

	Estimate	Std. Error	t value	Pr(>|t|)
(Intercept)	0.468231	0.003222	145.303	0
medicaid	-0.01246	0.008393	-1.48472	0.137619
year2	0.011801	0.003427	3.443598	0.000574
year3	0.007833	0.004405	1.778205	0.07537
year4	0.005021	0.004421	1.135807	0.256037
year5	-0.00869	0.004198	-2.07023	0.03843
year6	-0.01331	0.004377	-3.04011	0.002365
rural	0.011146	0.003397	3.281087	0.001034
car	0.046164	0.005636	8.190381	2.6E-16
psy	0.07055	0.002941	23.98664	3.8E-127
skc	0.028964	0.004405	6.575953	4.83E-11
cns	-0.0365	0.007664	-4.76257	1.91E-06
pul	0.035319	0.004426	7.980366	1.46E-15
gi	0.052558	0.004413	11.91021	1.05E-32
dia	-0.0007	0.010309	-0.06786	0.945898
skn	0.007261	0.005514	1.316981	0.187845
ren	0.020865	0.010088	2.068203	0.038621
can	0.009227	0.016749	0.550919	0.581689
dd	-0.02335	0.022538	-1.036	0.300202
gen	0.171664	0.004589	37.40386	3.4E-306
met	-0.00824	0.00792	-1.04019	0.298251
eye	-0.01037	0.019692	-0.52653	0.59852
cer	-0.0587	0.033784	-1.73755	0.08229
hem	0.086844	0.011598	7.487872	7E-14
medicaid:time	0.001447	0.003531	0.409728	0.682005
medicaid:intervention	-0.09794	0.018921	-5.17637	2.26E-07
medicaid:time:intervention	0.031989	0.005	6.397846	1.58E-10

© 2020 Rodriguez MI et al. *JAMA Network Open*.

eTable 6. Raw Model Output From Primary Comparative Interrupted Time Series Model for Women Ages 30-34

	Estimate	Std. Error	t value	Pr(>|t|)
(Intercept)	0.379495	0.002918	130.0753	0
medicaid	-0.06592	0.007861	-8.38501	5.07E-17
year2	0.019048	0.003003	6.343428	2.25E-10
year3	0.026731	0.00384	6.960861	3.38E-12
year4	0.018128	0.003953	4.586235	4.51E-06
year5	0.015094	0.003853	3.917412	8.95E-05
year6	0.013548	0.004028	3.363599	0.000769
rural	-0.00797	0.003542	-2.25047	0.024419
car	0.027919	0.004871	5.731359	9.96E-09
psy	0.050425	0.002887	17.46909	2.46E-68
skc	0.020261	0.003992	5.074899	3.88E-07
cns	-0.01477	0.006638	-2.22423	0.026133
pul	0.020236	0.004264	4.746066	2.07E-06
gi	0.040505	0.004212	9.617119	6.77E-22
dia	0.004303	0.008811	0.488422	0.625251
skn	-0.00103	0.005331	-0.19324	0.846768
ren	0.020709	0.008934	2.318027	0.020448
can	0.007154	0.013426	0.532817	0.59416
dd	0.028318	0.026421	1.071778	0.28382
gen	0.189299	0.004757	39.79183	0
met	-0.01422	0.007295	-1.94879	0.051321
eye	0.011613	0.016911	0.686692	0.492277
cer	-0.04256	0.029595	-1.43818	0.150383
hem	0.10387	0.010965	9.473257	2.71E-21
medicaid:time	0.004297	0.003255	1.320221	0.186761
medicaid:intervention	-0.0997	0.018006	-5.53726	3.07E-08
medicaid:time:intervention	0.037129	0.004724	7.85932	3.86E-15

© 2020 Rodriguez MI et al. *JAMA Network Open*.

eTable 7. Raw Model Output From Primary Comparative Interrupted Time Series Model for Women Ages 35-50

	Estimate	Std. Error	t value	Pr(>|t|)
(Intercept)	0.166058	0.001223	135.8311	0
medicaid	-0.03138	0.003818	-8.22128	2.01E-16
year2	0.014824	0.001127	13.15813	1.53E-39
year3	0.024384	0.00141	17.29463	5.16E-67
year4	0.031077	0.00151	20.5798	4.16E-94
year5	0.04332	0.001556	27.83194	1.8E-170
year6	0.053667	0.001628	32.95769	3.3E-238
rural	-0.01539	0.002049	-7.50895	5.96E-14
car	0.001067	0.001981	0.538848	0.589992
psy	0.043678	0.001641	26.62128	3.9E-156
skc	0.00095	0.00185	0.513223	0.607796
cns	-0.00983	0.00306	-3.21359	0.001311
pul	0.005069	0.002156	2.350589	0.018744
gi	0.012861	0.002054	6.261449	3.81E-10
dia	-0.03106	0.003115	-9.96951	2.07E-23
skn	-0.00411	0.002579	-1.59393	0.110951
ren	-0.0037	0.003739	-0.98987	0.322235
can	-0.04775	0.004125	-11.576	5.46E-31
dd	0.152126	0.024455	6.220747	4.95E-10
gen	0.188935	0.002958	63.86721	0
met	-0.00599	0.003341	-1.7933	0.072924
eye	-0.01832	0.005294	-3.46057	0.000539
cer	-0.04313	0.010711	-4.02712	5.65E-05
hem	0.013927	0.00577	2.413781	0.015788
medicaid:time	0.003328	0.001519	2.190461	0.028491
medicaid:intervention	-0.08704	0.009242	-9.41769	4.61E-21
medicaid:time:intervention	0.030997	0.002359	13.13974	1.95E-39

© 2020 Rodriguez MI et al. *JAMA Network Open*.

eFigure. CONSORT Diagram of Sample Selection

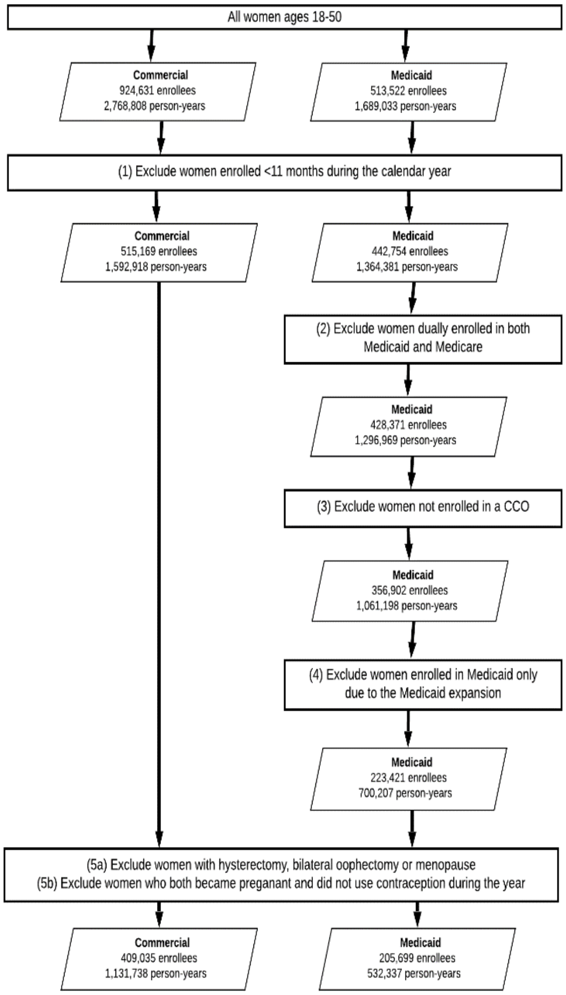

© 2020 Rodriguez MI et al. *JAMA Network Open*.
